# MS/MS-Based Molecular Networking Approach for the Detection of Aplysiatoxin-Related Compounds in Environmental Marine Cyanobacteria

**DOI:** 10.3390/md16120505

**Published:** 2018-12-13

**Authors:** Chi Ying Gary Ding, Li Mei Pang, Zhao-Xun Liang, Kau Kiat Kelvin Goh, Evgenia Glukhov, William H. Gerwick, Lik Tong Tan

**Affiliations:** 1 Natural Sciences and Science Education, National Institute of Education, Nanyang Technological University, Singapore 637616, Singapore; ding_gary@yahoo.com.sg; 2 School of Biological Sciences, Nanyang Technological University, Singapore 637551, Singapore; PANG0165@e.ntu.edu.sg (L.M.P.); ZXLiang@ntu.edu.sg (Z.-X.L.); 3 Singapore Phenome Centre, Lee Kong Chian School of Medicine, Nanyang Technological University, Singapore 636921, Singapore; kelvin.goh.kau.kiat@sgh.com.sg; 4 Center for Marine Biotechnology and Biomedicine, Scripps Institution of Oceanography, University of California at San Diego, La Jolla, CA 92093, USA; eglukhov@ucsd.edu (E.G.); wgerwick@ucsd.edu (W.H.G.)

**Keywords:** filamentous marine cyanobacteria, aplysiatoxin, molecular networking, *Trichodesmium erythraeum*, *Okeania* sp., *Oscillatoria* sp.

## Abstract

Certain strains of cyanobacteria produce a wide array of cyanotoxins, such as microcystins, lyngbyatoxins and aplysiatoxins, that are associated with public health issues. In this pilot study, an approach combining LC-MS/MS and molecular networking was employed as a rapid analytical method to detect aplysiatoxins present in four environmental marine cyanobacterial samples collected from intertidal areas in Singapore. Based on 16S-ITS rRNA gene sequences, these filamentous cyanobacterial samples collected from Pulau Hantu were determined as *Trichodesmium erythraeum*, *Oscillatoria* sp. PAB-2 and *Okeania* sp. PNG05-4. Organic extracts were prepared and analyzed on LC-HRMS/MS and Global Natural Product Social Molecular Networking (GNPS) for the presence of aplysiatoxin-related molecules. From the molecular networking, six known compounds, debromoaplysiatoxin (**1**), anhydrodebromoaplysiatoxin (**2**), 3-methoxydebromoaplysiatoxin (**3**), aplysiatoxin (**4**), oscillatoxin A (**5**) and 31-noroscillatoxin B (**6**), as well as potential new analogues, were detected in these samples. In addition, differences and similarities in molecular networking clusters related to the aplysiatoxin molecular family were observed in extracts of *Trichodesmium erythraeum* collected from two different locations and from different cyanobacterial species found at Pulau Hantu, respectively.

## 1. Introduction

Marine filamentous cyanobacteria are prolific sources of novel secondary metabolites, and a number of these compounds possess significant biological activity, having agricultural and pharmaceutical applications [1,2]. A notable example is a series of highly cytotoxic lead compounds belonging to the dolastatin class of molecules [3]. For instance, dolastatin 10 and synthetic derivatives, soblidotin (TZT-1027) and tasidotin, entered clinical trials in the 1990s as anticancer agents but dropped out in Phase II due to toxicity and/or lack of efficacy in trials [4]. A synthetic analogue, monomethyl auristatin E, was subsequently formulated as an antibody-drug conjugate, brentuximab vendotin (Adecetris^®^), for the treatment of Hodgkin’s lymphoma and systemic anaplastic large cell lymphoma in 2012 [5].

In recent years, various studies revealed that certain marine cyanobacterial strains produce compounds that concurrently provide beneficial and detrimental effects to human health. One such compound is the polyketide-based cyanotoxin, aplysiatoxin. Cyanotoxins collectively cause a wide spectrum of health conditions, ranging from dermatitis to severe carcinoma [6,7,8]. Aplysiatoxin and its related compounds, which includes oscillatoxin A and nhatrangins, have been reported from *Lyngbya majuscula*, *Oscillatoria nigro-viridis* and *Trichodesmium erythraeum* [6]. The tumor promoting activity of aplysiatoxin and debromoaplysiatoxin is attributed to their specific molecular roles as protein kinase C activators. Interestingly, a simplified synthetic analogue of aplysiatoxin, aplog-1, was recently reported to possess anti-cancer properties with mode of action similar to bryostatin 1 [9,10,11,12,13]. In addition, studies revealed several aplysiatoxin-related compounds to display therapeutic activities, such as anti-Chikungunya and modulation of potassium ion channels [14,15]. A recent study by Richard et al. demonstrated that aplysiatoxin and its debrominated analogue induced expression of latent HIV-1 provirus in both cell line and primary cell models [16]. 

Due to the varied biological activities of the aplysiatoxins, they make good candidates for biomedical research as well as in their detection and monitoring of toxins during algal bloom occurrence. Given the correlation between global warming and occurrence of cyanobacterial blooms, the incidence of illness caused by contact with cyanotoxins can potentially be increased [8]. In contrast to research on hepatotoxins (e.g., microcystins and nodularin) as well as other neurotoxins (e.g., β-N-methylamino-l-alanine), very little work has been conducted on the development of detection methods of other cyanotoxins, such as the aplysiatoxins [17]. In this report, we employed MS/MS-based molecular networking approach for the rapid detection of aplysiatoxin class of molecules from environmental marine cyanobacterial samples obtained from four intertidal areas in Singapore. The detection method, based on either HPLC-HRMS/MS or UPLC-HRMS/MS combined with data analysis using Global Natural Product Social Molecular Networking (GNPS), led to the detection of both known and new analogues related to the aplysiatoxin class of cyanotoxins.

## 2. Results

### 2.1. Phylogenetic Analysis of Environmental Marine Cyanobacterial Samples

**Figure 1:**
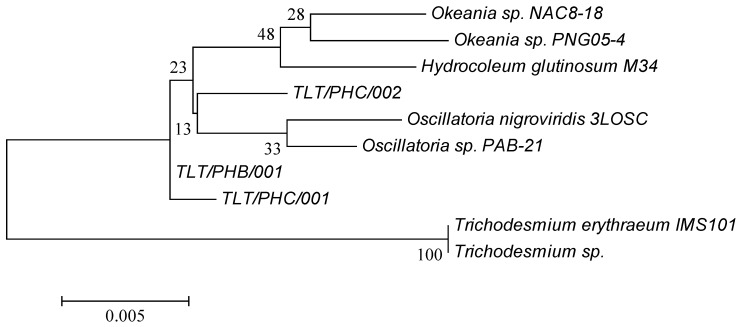
Phylogenetic tree (maximum likelihood) for three environmental marine cyanobacterial strains, TLT/PHB/001, TLT/PHC/001 and TLT/PHC/002. The scale bar indicates 0.005 expected nucleotide substitutions per site.

Of the four marine cyanobacterial samples analyzed in this study, three samples were collected between June and August 2016 at three intertidal areas located at Pulau Hantu Besar (TLT/PHB/001), Pulau Hantu Central Lagoon area 1 (TLT/PHC/001) and Pulau Hantu Central Lagoon area 2 (TLT/PHC/002). A fourth cyanobacterial sample, *Trichodesmium erythraeum*, was previously collected from Pulau Seringat Kias (TLT/PSK/001) in August 2012 [8]. A phylogenetic tree comprising of the three cyanobacterial samples from Pulau Hantu was constructed against seven cyanobacterial strains and is presented in Figure 1. Gene sequence data, obtained after amplification of the 16S rRNA gene, for TLT/PHB/001, TLT/PHC/001 and TLT/PHC/002 was assigned to *Trichodesmium erythraeum* with a 97.8% sequence identity (1465 bp, 100% gene sequence coverage), *Okeania sp.* PNG05-4 with a 98.8% sequence identity (1399 bp, 95.8% gene sequence coverage), and *Oscillatoria sp.* PAB-21 with a 99.1% sequence identity (1363 bp, 93.4% gene sequence coverage), respectively. Individual score and coverage/identity giving corresponding closest homologs based on 16S-ITS rRNA sequences from TLT/PHB, TLT/PHC001 and TLT/PHC002 were tabulated in Table S1 (Supplementary Data), and complete 16S rRNA sequence in Table S2 (Supplementary Data).

### 2.2. MS/MS-Based Molecular Networks of Marine Cyanobacterial Extracts

**Figure 2:**
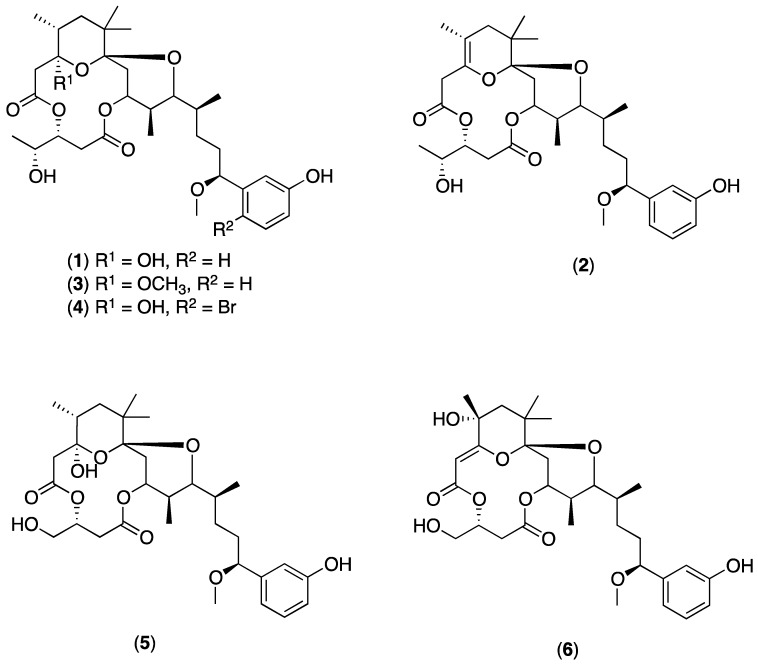
Chemical structures of aplysiatoxin (**4**), and related analogues debromoaplysiatoxin (**1**), anhydrodebromoaplysiatoxin (**2**), 3-methoxydebromoaplysiatoxin (**3**), oscillatoxin A (**5**) and 31-noroscillatoxin B (**6**).

**Figure 3:**
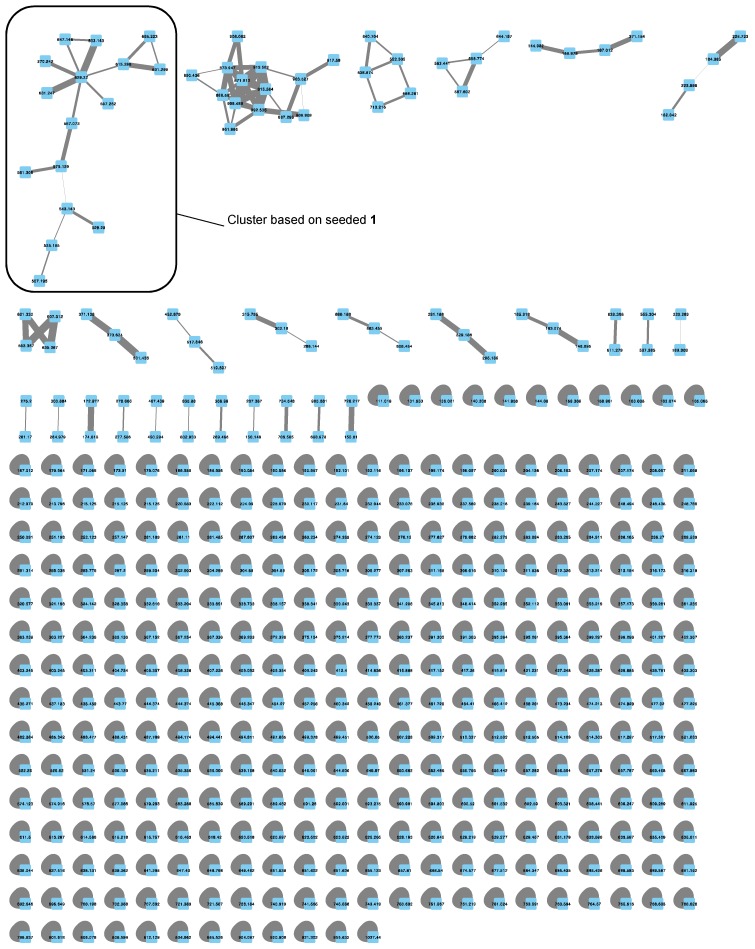
Molecular network of the extract obtained from TLT/PHB/001 generated using Cytoscape v3.5.1. (The Cytoscape Consortium, New York, NY, USA).

**Figure 4:**
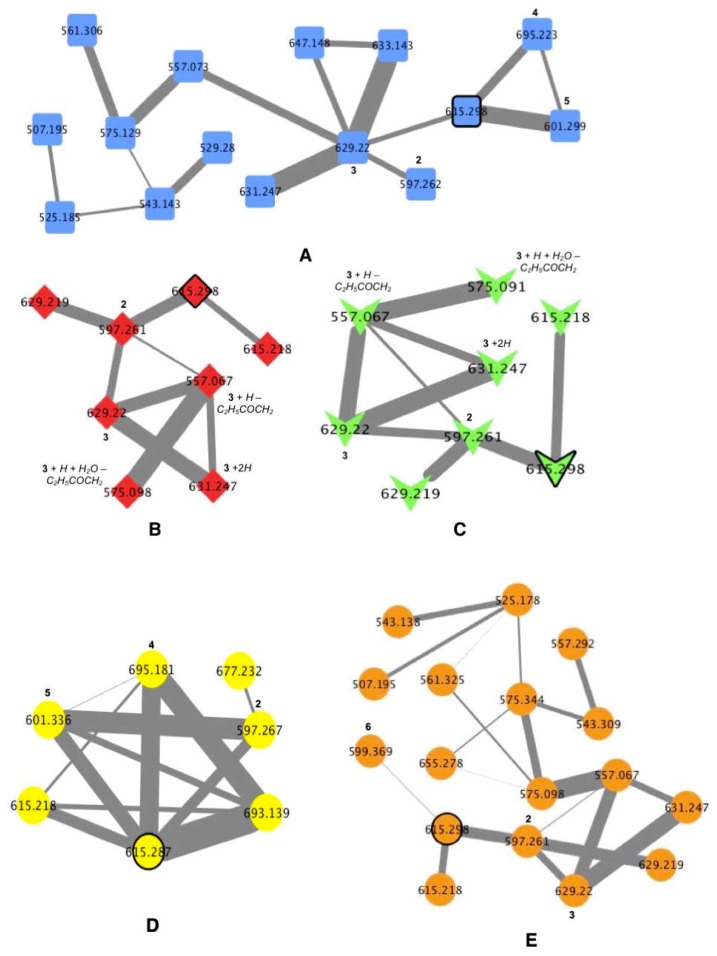
Cyanobacterial molecular network clusters based on the seeded **1** (nodes outlined) with a cosine similarity cutoff of 0.65 obtained from extracts. (**A**): TLT/PHB/001 (square nodes), (**B**): TLT/PHC/001 (diamond nodes), (**C**): TLT/PHC/002 (V-shape nodes), (**D**): TLT/PSK/001a (ellipse nodes; based on Thermo–Finnigan system) and (**E**): TLT/PSK/001b (ellipse nodes; based on Waters system). Italicized labels indicate putative assignments; numbers in bold indicate compounds **1** to **6** (refer to Figure 2). Edge thickness corresponds to relative cosine similarity between nodes.

Organic crude extracts from each of the cyanobacterial samples were prepared and analyzed initially by UPLC–HRMS/MS Waters system (Milford, MA, USA). In addition, organic crude extract from TLT/PSK/001 were analyzed on both HPLC-HRMS/MS Thermo–Finnigan system (Waltham, MA, USA) (TLT/PSK/001a) and UPLC-HRMS/MS Waters system (TLT/PSK/001b). Mass spectrometric data obtained from these analyses were then used to generate molecular networking clusters using the online platform Global Natural Products Social Molecular Networking (GNPS). Debromoaplysiatoxin (**1**) (Figure 2), isolated from the extract of TLT/PHC/001, was used as seed molecule in the analysis (HRESIMS of sodiated molecular ion *m*/*z* 615.3005, calculated for C_32_H_48_O_10_Na^+^ 615.2987, difference = 2.9 ppm). Molecular networks of the extracts (an example is shown for TLT/PHB/001 in Figure 3), visualized using the software Cytoscape v3.5.1 (The Cytoscape Consortium, New York, NY, USA), revealed the presence of clusters related to the molecular family of the aplysiatoxin class in all four environmental cyanobacterial samples (clusters **A** to **E** in Figure 4). In addition to the presence of the seeded debromoaplysiatoxin, five other related compounds (Figure 2), namely, anhydrodebromoaplysiatoxin (**2**), 3-methoxydebromoaplysiatoxin (**3**), aplysiatoxin (**4**), oscillatoxin A (**5**) and 31-noroscillatoxin B (**6**), were detected in clusters **A**, **B**, **C** and **E** based on their HRMS (High-Resolution Mass Spectrometry) data (Table 1 and Figure 4). Furthermore, potential new aplysiatoxin analogues, based on their relatedness to **1**, were detected from the clusters. These potential new compounds are indicated as 15 nodes from cluster **A**, eight nodes from both cluster **B** and **C**, seven nodes from cluster **D** and 17 nodes from cluster **E** (Figure 4). Some putative assignments for the new aplysiatoxin analogues were included, based on the HRMS *m*/*z* values. 

## 3. Discussion

Certain cyanobacteria strains are known to produce a wide array of toxic metabolites or cyanotoxins, such as aplysiatoxins, microcystins and nodularins [7]. Many of these cyanotoxins affect human health through their actions as tumor promoters, diarrhetic, and pro-inflammatory activities [18]. In addition, these metabolites may also limit various aquatic recreational activities, thereby affecting related economies. Such impacts are particularly acute in certain geographical regions, such as Singapore, where its location in the tropics provides a conducive environment for the occurrence, growth, and more importantly blooms of marine cyanobacteria [19]. For instance, regular occurrence of marine cyanobacterial blooms has been sighted at several offshore islands in Singapore, including Pulau Semakau, P. Seringat-Kias, P. Hantu, P. Ubin and P. Sakijang Bendera. The cyanobacterial samples used in this study were obtained from islands designated as tourist destinations with aquatic recreational activities, including SCUBA diving and swimming, as the main attraction. The persistent occurrence of marine cyanobacteria at these islands (personal observation by Lik Tong Tan) warrants setting up of a rapid and relatively straightforward analytical method for the detection of cyanotoxins from environmental samples. A rapid detection method is integral and equally imperative to prevent the general public’s exposure to these toxic compounds.

In this study, molecular networking clusters of various environmental marine cyanobacterial extracts were analyzed using a MS/MS-based molecular networking approach [20]. Initial chemical investigation of the organic extract obtained from the cyanobacterial sample TLT/PHC/001 collected at Pulau Hantu resulted in the isolation of the known cyanotoxin debromoaplysiatoxin (**1**). This molecule was subsequently used as a seed molecule for the creation of the aplysiatoxin molecular family in the molecular network. Each node within the clusters represent an ionized compound and dereplication was performed by comparison with the *m*/*z* values of known aplysiatoxin-related analogues (Table 2).

In addition to debromoaplysiatoxin, a number of known analogues, anhydrodebromoaplysiatoxin (**2**), 3-methoxydebromoaplysiatoxin (**3**), aplysiatoxin (**4**), oscillatoxin A (**5**) and 31-noroscillatoxin B (**6**), were found to be present in the molecular network clusters **A**, **B**, **C** and **E**. The identity of these known compounds was confirmed by comparing the calculated *m*/*z* values with that obtained from HRMS/MS (differences of less than 5 ppm) as shown in Table 1 and Table 2. Compounds **1**–**3** were present in clusters **A**, **B**, **C** and **E**. Additional compounds **4** and **5** were also present in cluster **A** while **6** was found in cluster **E**.

**Figure 5:**
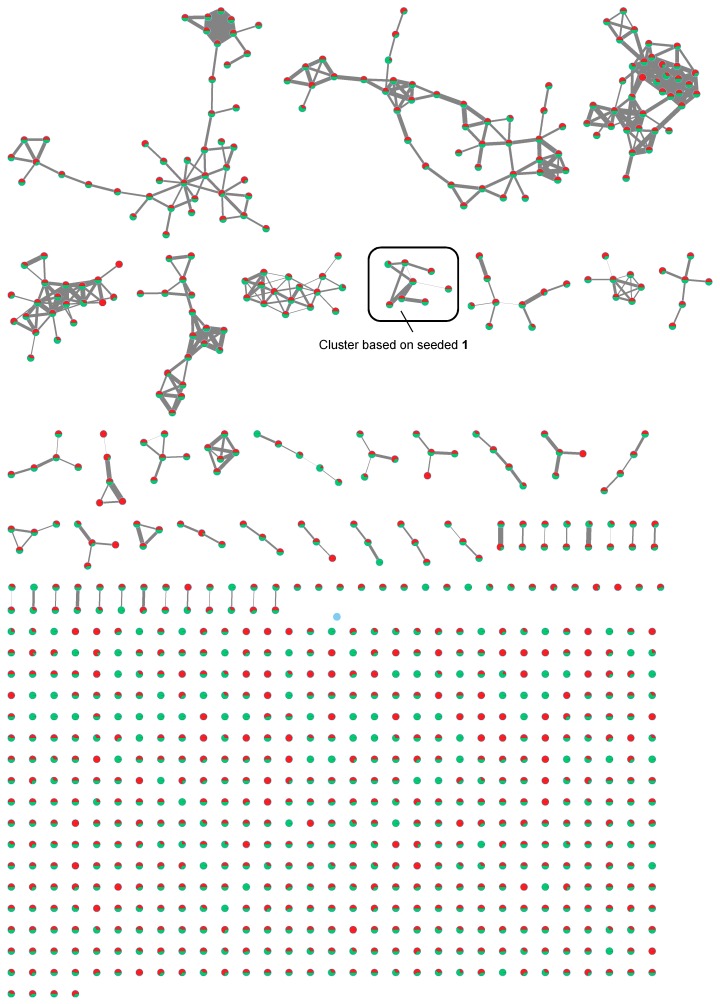
Overlaying of two molecular networks based on the extracts obtained from TLT/PHC/001 and TLT/PHC/002. Red and green nodes represent compounds produced by TLT/PHC/001 and TLT/PHC/002, respectively. Relative proportion of the compound belonging to each extract was represented as a pie chart in each node.

The phylogenetic results of the cyanobacterial samples, coupled with careful analysis of the molecular networking clusters of their extracts, revealed a number of interesting observations. Though both TLT/PHB/001 and TLT/PSK/001 cyanobacterial samples have been identified as *Trichodesmium erythraeum*, their clusters (**A** and **E**), related to the aplysiatoxin molecular family based on the seeded **1**, were different (Figure 4). Clusters **A** and **E** gave 15 and 17 nodes, respectively, both revealing the presence of all three compounds **1**–**3**. The difference in the complexity of these two *Trichodesmium erythraeum*-derived molecular network clusters and the number of nodes present may be attributed to the versatility of the biosynthetic gene clusters present in the same cyanobacteria, and different environmental triggers present in the two locations where the cyanobacteria were collected. On the other hand, aplysiatoxin-related clusters **B** and **C** produced from two different cyanobacterial species, TLT/PHC/001 (identified as *Okeania sp.* PNG05-4) and TLT/PHC/002 (identified as *Oscillatoria sp.* PAB-21), respectively, with only slight difference in the *m*/*z* (within 5 ppm) observed between corresponding nodes. Interestingly, these two samples were collected from sites that were just 100 m apart at Pulau Hantu. In addition, when comparing the full molecular networks of these two different cyanobacterial species (Figure 5), minimal differences in the compounds, aside from the aplysiatoxin-related cluster, were observed. These observations suggest that similar compounds are largely produced as a result of the same environmental triggers, though by different cyanobacteria, and this can be validated with the help of results obtained through the investigations of related biosynthetic gene clusters of TLT/PHC/001 and TLT/PHC/002 in producing the aplysiatoxin-related compounds. 

It has been shown that the MS/MS-based molecular networking is useful for dereplication of known compounds as well as the discovery of potentially new natural products [21]. In molecular networking, MS/MS spectra are compared based on the similarity and differences in fragmentation patterns. Structurally related compounds can then be clustered as nodes due to certain degrees of similarity between the compounds expressed as edges in the molecular network. In addition, the availability of HRMS/MS spectral data of known cyanotoxins in GNPS database could facilitate the detection process [22]. Recently, Teta and co-workers used a similar MS-based molecular networking approach to detect new microcystin variants from the 2014 freshwater cyanobacterial harmful bloom at Green Lake, Seattle [23]. In another study by Briand et al., molecular networking was employed to track changes in secondary metabolic profiles, including microcystins and other peptides, of *Microcystis aeruginosa* strains due to intraspecific interactions [24]. In this present study, two nodes with *m*/*z* 629.219 and 629.220 observed in clusters **B**, **C** and **E** are likely to be compound **3**. However, it is evident that only the node with observed value of 629.22 fits within the accepted ppm difference for compound **3** (Figure 4). Considering the presence of both nodes found within the same cluster, the node of *m*/*z* 629.219 could be a new analogue related to **2**. A similar relationship was observed from the nodes of *m*/*z* 615.218 and 615.298 present in the same set of clusters (Figure 4). Some putative assignments were provided to unknown compounds based on observed HRMS of the nodes within each cluster. An example is the compound with *m*/*z* value of 631.247 in clusters **B** and **C** that could correspond to an analogue of **3** having a reduced carbonyl moiety or cleavage to the oxide functionality of either tetrahydropyran rings.

The advantages of use and advancements in instrumentation provided a variety of MS/MS systems used by laboratories today. An interesting technical approach adopted in this study was to employ two different systems in the analysis, namely a low-resolution Thermo-Finnigan HPLC-MS/MS and a high-resolution Waters UPLC-MS/MS. The main reason was to make comparisons of the molecular networks created based on each system. The organic extract from TLT/PSK/001 was randomly chosen and molecular networks were then constructed individually using the MS/MS data acquired from both MS systems using similar parameters. Analysis of the molecular network clusters TLT/PSK/001a (**D**) and TLT/PSK/001b (**E**) using Thermo–Finnigan and Waters systems, respectively, revealed several differences. The first difference was the size of each molecular network due to a difference in quantity of nodes present, with 7 and 17 nodes in clusters **D** and **E**, respectively. Another difference was observed comparing compounds **1**, **2** and **6** present in both clusters, where each corresponding node in both clusters gave different *m*/*z* values. A case in point is the node that corresponds to **2**, with *m*/*z* of 597.267 and 597.261 observed in clusters **D** and **E**, respectively. The chemical identity of the corresponding nodes was achieved and confirmed to be **2** in both clusters, where a mass difference of within 5 ppm was determined based on the observed and calculated *m*/*z* obtained from its respective system. The last difference was the detection of different compounds from the same cyanobacterial strain in the two molecular networks. Compounds **4** and **5** were observed exclusively in cluster **D** obtained from the Thermo–Finnigan system; compound **3** observed in cluster **E** created using the Waters system. While the exact explanation could not be provided, these differences between the clusters may be attributed largely to the different MS instruments’ operating parameters (i.e., resolution, type of ionization and detector), hence care should be taken while comparing molecular networks from different systems.

Current aquatic biomonitoring and ecotoxicology programs in Singapore are confined to the analysis of freshwater cyanobacterial strains for hepatoxins, such as microcystins and nodularin. Methods, such as mass spectrometry, capillary electrophoresis and qPCR, have been used for the detection of microcystins as well as for assessing cyanotoxin risk in freshwater cyanobacterial strains obtained from reservoirs in Singapore [25,26,27]. The unprecedented nature of this pilot study underscores the importance of the MS/MS-based molecular networking approach as a rapid analytical technique to detect non-peptidic cyanotoxins, such as the aplysiatoxins, from environmental samples. Moreover, recent reports showed that several aplysiatoxin analogues possess therapeutic activities, such as antiviral and potassium channel inhibition activities [14,15]. In light of these discoveries, the MS/MS-based molecular networking approach employed in this study could aid in the detection of new aplysiatoxin analogues that harbors varied biomedical potential, contributing to the growing list of marine natural products as therapeutic agents.

## 4. Materials and Methods 

### 4.1. General Experimental Procedures

Proton NMR spectra were recorded in CDCl_3_ on a 400 MHz Bruker (Billerica, MA, USA) NMR spectrometer using the residual solvent signals (δ_H_ at 7.28 ppm) as internal standards. High-performance liquid chromatography isolation and purification of compound **1** was conducted on a Shimadzu (Nakagyo-ku, Kyoto, Japan) LC-8A preparative LC coupled to a Shimadzu SPD-M10A VP diode array detector. The UPLC-HRMS/MS analysis was conducted on a Waters (Milford, MA, USA) Xevo G2-XS qTOF with an ESI positive ion mode and data-dependent acquisition mode to obtain MS and MS/MS data. The HPLC-MS/MS analysis was conducted on a Thermo–Finnigan (San Jose, CA, USA) LCQ Advantage ion trap mass spectrometer with an ESI positive ion mode and data-dependent manner to obtain MS and MS/MS data. High-performance liquid chromatography preparative and analytical columns was obtained from Phenomenex (Torrance, CA, USA), and a UPLC analytical column was obtained from Waters. All solvents were HPLC-grade purchased from Thermo-Fisher Scientific (Waltham, MA, USA) and HPLC-grade water obtained by filtration using a Milli-Q Direct water purification system from Millipore (Billerica, MA, USA) unless otherwise stated. Deuterated NMR solvents were purchased from Cambridge Isotope Laboratories (Tewksbury, MA, USA).

### 4.2. Marine Cyanobacterial Collections

Samples of the benthic filamentous marine cyanobacteria were collected from offshore islands of Pulau Seringat Kias on August 7, 2012 (TLT/PSK/001), Pulau Hantu Besar, on 9 June 2016 (TLT/PHB/001), and Pulau Hantu Central Lagoon area 1 on 6 August 2016 (TLT/PHC/001) and Pulau Hantu Central Lagoon area 2 on 9 June 2016 (TLT/PHC/002). Voucher samples are stored at Natural Products Chemistry Laboratory, National Institute of Education, Nanyang Technological University, Singapore as TLT/PSK/07AUG2012/001, TLT/PHB/09JUNE2016/001, TLT/PHC/06AUG2016/001 and TLT/PHC/09JUNE2016/002, respectively. A small amount of the cyanobacterial samples collected in 2016 (TLT/PHB/001, TLT/PHC/001 and TLT/PHC/002) was preserved for genetic analysis in RNAlater^®^ (Ambion, Austin, TX, USA). Chemistry samples were preserved in 70% aqueous EtOH at −20 °C before chemical extraction.

### 4.3. DNA Isolation and PCR Amplification

Genetically preserved biomass (~ 150 mg) of each of the three filamentous marine cyanobacteria samples were washed 3 times with 3M NaCl solution. Genomic DNA was extracted using CTAB procedure as described previously, with increased lysozyme concentration of 5 mg/mL. The 16S rRNA genes and the 16S-23S ITS regions were PCR-amplified using primers 27F (5′-AGAGTTTGATCMTGGCTCAG-3′) and 340 (5′-CTCTGTGTGCCTAGGTATCC-3′) [28,29]. The PCR reaction volumes were 25 µL containing: 1 µL (~100 ng) of DNA, 5 µL of 5 × Q5 reaction buffer, 0.5 µL of (10 mM) dNTP mix, 1.25 µL of each primer (10 µM), 0.25 µL of Q5 High-Fidelity DNA polymerase and 15.75 µL of dH_2_O. The PCR reactions were performed in a T100^TM^ Thermal Cycler (Bio-Rad, Hercules, CA, USA) using the following program: Initial denaturation for 30 s at 98 ^°^C, amplification by 30 cycles of 10 s at 98 °C, 15 s at 63 °C and 2 min at 72 °C, and final elongation for 10 min at 72 °C. The PCR products were analyzed on 1% agarose gel in TAE (Tris-acetate-EDTA) buffer and visualized by gel red. The PCR products were purified using NucleoSpin Gel and PCR Clean-up kit (Macherey-Nagel, Duren, Germany). All purified PCR products of 27F and 340 primers were sequenced with 883R primer (5′-ATTAAACCACATACTCCACC-3′), FR primer (5′-ACGAGCTGACGACAGCCATG-3′), HR primer (5′-AAGGAGGTGATCCAGCCGCA-3′) and 1052F (5′-CATCATTAAGTTGGGCACTC-3′) [22]. Sequencing was performed at 1^st^BASE Laboratories Sdn BhD (Singapore).

### 4.4. Phylogenetic Analysis

Gene sequences were aligned using MUSCLE (European Bioinformatics Institute, EMBL-EBI, Cambridge, United Kingdom) [30]. The phylogenetic tree was plotted using maximum likelihood (ML) in MEGA, v6 (Pennsylvania State University, State College, PA, USA) [31]. The 16S rRNA gene sequence of each cyanobacterial sample was multiply aligned with 7 cyanobacterial strains obtained from the GenBank/EMBL/DDBJ databases. Bootstrap re-sampling was performed with 1000 replicates.

### 4.5. Extraction of Crude Extracts and Isolation of Debromoaplysiatoxin (1)

Each of the four environmental cyanobacterial samples (about 10 g each) were exhaustively extracted with 2:1 CH_2_Cl_2_/MeOH. The crude extract from TLT/PHC/001 (1.421 g) was chosen and dried under vacuum and applied to a silica gel VLC column. The column was eluted to produce nine sub-fractions: C1 (elution by 100% hexanes), C2 (elution by 10% EtOAc/hexanes), C3 (elution by 30% EtOAc/hexanes), C4 (elution by 50% EtOAc/hexanes), C5 (elution by 60% EtOAc/hexanes), C6 (elution by 80% EtOAC/hexanes), C7 (elution by 100% EtOAc), C8 (elution by 10% MeOH/EtOAc) and C9 (20% MeOH/EtOAc). Sub-fractions were subjected to Brine Shrimp Toxicity Assay and sub-fraction C6 displayed mortality at 93.2% and 81.9% when tested at 100 ppm and 10 ppm, respectively. The remaining sub-fraction C6 (121.4 mg) was filtered over a C_18_ SPE cartridge by elution with 50% MeOH/H_2_O. The filtered sample (29.0 mg) was subjected to preparative reversed-phase HPLC separation using a Phenomenex Luna phenyl-hexyl column (250 mm × 100 mm, 5 μm). Elution at 3.0 mL/min using eluent A: H_2_O; eluent B: CH_3_CN; gradient: 38% B for 1.5 min, 38→80% B in 5.5 min, 80→100% B in 5 min, then held at 100% B for 18 min to yield **1** (8 mg, *t*_R_ = 20.024 min). Structure and identity of **1** was confirmed based on proton NMR in CDCl_3_ (Figure S1, Supplementary Data) and HRESIMS *m*/*z* 615.3005 (calculated for C_32_H_48_O_10_Na^+^, 615.2987, difference = 2.9 ppm). Mass spectrometry parameters included a spray voltage of 3 kV, cone voltage of 40 V, source temperature of 120 °C, desolvation temperature of 450 °C, cone gas flow of 50 L/h and desolvation gas flow of 950 L/h.

### 4.6. UPLC-HRMS/MS Analysis

Crude extracts were re-dissolved in 1 mL CH_3_CN with vortex mixing over 5 min before transferring into separate Eppendorf tubes. Tubes were then centrifuged at 10,000 rpm at 4 °C over 10 min and the supernatant was aliquoted and finally diluted with CH_3_CN to 10,000× dilution. Then, 1.5 μL of each 10,000× dilution sample was subjected to UPLC-HRMS/MS performed using the same high-resolution spectrometer and parameters stated above, together with a Waters ACQUITY BEH C_18_50 mm × 2.1 mm, 1.7 μm column and maintained at a column temperature of 40 °C and sample temperature at 4 °C using a step elution program of ‘1’ based on the Waters system. Step elution program was as follows: Mobile phase of 98% CH_3_CN in 0.1% aq. HCOOH/0.1% aq. HCOOH, run time of 14 min and flow rate of 0.5 mL/min. All the mass spectra were recorded in the positive-ion mode. Data were collected in the data-dependent acquisition mode, in which the first ten most intense ions of a full-scan mass spectrum were subjected to MS/MS analysis. Tandem mass spectrometry scans were obtained for selected ions with a mass range of 100–2000 Da, MS scan time of 0.33 s over 12 min, MS/MS scan time of 0.10 s and a collision energy ramp of 10–50 V.

### 4.7. HPLC-MS/MS Analysis

TLT/PSK/001 was re-dissolved in MeOH and 20 μL of the solution was subjected (no-waste mode) to HPLC-MS/MS using a Thermos–Finnigan system with a Phenomenex Kinetex C _18_ 100 mm × 4.6 mm, 5 μm column. Elution at 0.5 mL/min using eluent A: 0.1% aq. HCOOH in H_2_O; eluent B: CH_3_CN; gradient: 30→100% B over 20 min, then held at 100% B for 10 min. The HPLC eluate was electrospray ionized at 35 eV and analyzed for positive ions, and MS/MS spectra were obtained in a data-dependent manner using collision induced dissociation (CID) at 35 eV.

### 4.8. Molecular Networking Creation

The mass spectral data were converted to .mzXml format using the program msconvert (ProteoWizard Software Foundation, Los Angeles, CA, USA). A molecular network was created using the online workflow at GNPS website [32]. The data were filtered by removing all MS/MS peaks within ± 17 Da of the precursor *m*/*z*. Tandem mass spectrometry spectra were window filtered by choosing only the top 6 peaks in the ± 50 Da window throughout the spectrum. The data were then clustered with MS-Cluster with a parent mass tolerance of 2.0 Da and a MS/MS fragment ion tolerance of 0.5 Da to create consensus spectra. Further, consensus spectra that contained less than 2 spectra were discarded. A network was then created where edges were filtered to have a cosine score above 0.65 and more than 10 matched peak. Further edges between two nodes were kept in the network if, and only if, each of the nodes appeared in each other’s respective top 10 most similar nodes. The spectra in the network were then searched against GNPS’ spectral libraries. The library spectra were filtered in the same manner as the input data. The data were then imported into Cytoscape v3.5.1 (The Cytoscape Consortium, New York, NY, USA) and displayed as a network of nodes and edges [26]. All matches kept between network spectra and library spectra were required to have a score above 0.65 and at least 1 matched peak. Analogue search was enabled against the library with a maximum mass shift of 100.0 Da.

## Figures and Tables

**Table 1:** Mass differences (**Δ**) of observed and calculated *m*/*z* values of known compounds found in clusters, based on HRMS data obtained from Waters system.

Cluster	Compound	Molecular Formula of Observed [M + Na]^+^	Calculated *m*/*z*	Observed *m*/*z*	*^a^*Δ (ppm)
**A**	**1**	C_32_H_48_O_10_Na	615.2987	615.2988	+0.1
	**2**	C_32_H_46_O_9_Na	597.3040	597.3047	+1.1
	**3**	C_33_H_50_O_10_Na	629.3302	629.3308	+0.9
	**4**	C_32_H_47_O_10_BrNa	695.2236	695.2227	−0.4
	**5**	C_31_H_46_O_10_Na	601.2989	601.2982	−1.2
**B**	**1**	C_32_H_48_O_10_Na	615.2987	615.3005	+2.9
	**2**	C_32_H_46_O_9_Na	597.3040	597.3024	−2.7
	**3**	C_33_H_50_O_10_Na	629.3302	629.3307	+0.8
**C**	**1**	C_32_H_48_O_10_Na	615.2987	615.2986	−0.1
	**2**	C_32_H_46_O_9_Na	597.3040	597.3058	+3.0
	**3**	C_33_H_50_O_10_Na	629.3302	629.3290	−1.9
**E**	**1**	C_32_H_48_O_10_Na	615.2987	615.2985	−0.3
	**2**	C_32_H_46_O_9_Na	597.3040	597.3027	−2.1
	**3**	C_33_H_50_O_10_Na	629.3302	629.3308	+0.9
	**6**	C_31_H_44_O_10_Na	599.2832	599.2843	+1.8

*^a^*Δ = Observed *m*/*z* − Calculated *m*/*z*.

**Table 2:** A list of known aplysiatoxin and related analogues isolated from marine cyanobacteria with the *m*/*z* values of commonly occurring proton and sodium adducts [1,7,14,15,16].

Compound	Molecular Formula	Molecular Mass	Calculated *m*/*z* [M + H]^+^	Calculated *m*/*z* [M + Na]^+^
Aplysiatoxin	C_32_H_47_O_10_Br	671.621	672.622	694.604
Debromoaplysiatoxin	C_32_H_48_O_10_	592.725	593.727	615.709
Oscillatoxin A	C_31_H_46_O_10_	578.698	579.700	601.682
Anhydro-19-bromoaplysiatoxin	C_32_H_44_O_9_Br_2_	732.502	733.502	755.484
Anhydro-19,21-dibromoaplysiatoxin	C_32_H_43_O_9_Br_3_	811.398	812.398	834.380
17-Bromooscillatoxin A	C_31_H_45_O_10_Br	657.594	658.595	680.577
17,19-Dibromooscillatoxin A	C_31_H_44_O_10_Br_2_	736.491	737.491	759.473
19-Bromoaplysiatoxin	C_32_H_46_O_10_Br_2_	750.517	751.518	773.500
Oscillatoxin B1	C_32_H_46_O_10_	590.709	591.711	613.692
Oscillatoxin B2	C_32_H_46_O_10_	590.709	591.711	613.692
31-Noroscillatoxin B	C_31_H_44_O_10_	576.683	577.684	599.666
Oscillatoxin D	C_31_H_42_O_8_	542.668	543.670	565.651
3-Methoxyaplysiatoxin	C_33_H_49_O_10_Br	685.641	686.648	709.639
3-Methoxydebromoaplysiatoxin	C_32_H_46_O_10_	606.745	607.753	629.735
Nhatrangin A	C_21_H_32_O_8_	412.474	413.482	435.465
Nhatrangin B	C_21_H_31_O_8_Br	491.370	492.378	514.360
Anhydrodebromoaplysiatoxin	C_32_H_46_O_9_	574.702	575.711	597.693
30-Methyloscillatoxin D	C_32_H_44_O_8_	556.695	557.696	579.678
Manauealide A	C_32_H_47_O_10_Cl	627.168	628.171	650.153
Manauealide B	C_32_H_47_O_10_Br	671.621	672.622	694.604
Manauealide C	C_34_H_50_O_11_	634.762	635.763	657.745
Anhydrooscillatoxin A	C_31_H_44_O_9_	560.683	561.685	583.667
Anhydroaplysiatoxin	C_32_H_45_O_9_Br	653.606	654.607	676.589
Neo-debromoaplysiatoxin A	C_32_H_46_O_10_	590.703	591.712	613.693
Neo-debromoaplysiatoxin B	C_27_H_38_O_6_	458.588	459.596	481.578

## Supplementary Materials

The following are available online at http://www.mdpi.com/1660-3397/16/12/505/s1, ^1^H NMR of **1**, and phylogenetic data of TLT/PHB/001, TLT/PHC/001 and TLT/PHC/002.
