# Supplementary material for: MS/MS-Based Molecular Networking Approach for the Detection of Aplysiatoxin-Related Compounds in Environmental Marine Cyanobacteria
Source: Mar Drugs. 2018 Dec 13;16(12):505. doi: 10.3390/md16120505 (PMC6315786; doi:10.3390/md16120505)
Supplement: Supplementary file 1 [file marinedrugs-16-00505-s001.pdf]

MS/MS-Based Molecular Networking Approach for the Detection of Aplysiatoxin-Related Compounds in Environmental Marine Cyanobacteria

Chi Ying Gary Ding [1], Li Mei Pang [2], Zhao Xun Liang [2], Kau Kiat Kelvin Goh [3], Evgenia Glukhov [4], William H. Gerwick [4] and Lik Tong Tan [1,*]

[1] Natural Sciences and Science Education, National Institute of Education, Nanyang Technological University, 1 Nanyang Walk, Singapore 637616
[2] School of Biological Sciences, Nanyang Technological University, Singapore 637551
[3] Singapore Phenome Centre, Lee Kong Chian School of Medicine, Nanyang Technological University, Singapore 636921
[4] Center for Marine Biotechnology and Biomedicine, Scripps Institution of Oceanography, University of California at San Diego, La Jolla, CA 92093, USA

SUPPLEMENTARY DATA

Figure **Page**

Figure		Page
Figure S1.	^{1}H spectrum of debromoaplysiatoxin (**1**) recorded in $CDCl_3$ at 400 MHz	2

Tables **Page**

Tables		Page
Table S1.	Partial 16S-ITS rRNA gene match for cyanobacterial strains	3
Table S2.	Complete 16S rRNA sequence of cyanobacterial strains	3

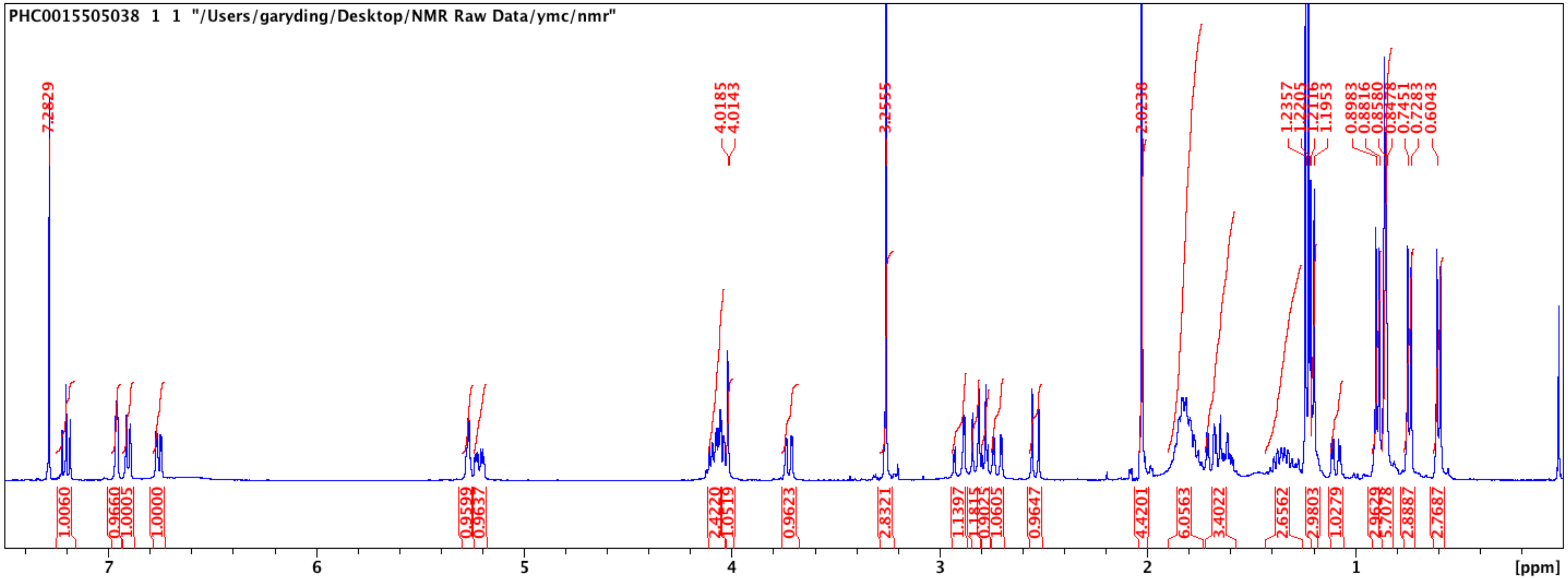

Figure S1. ^{1}H spectrum of debromoaplysiatoxin (**1**) recorded in $CDCl_3$ at 400 MHz

Table S1: Cyanobacterial strains partial 16s-ITS rRNA gene and its closet homologues in the nucleotide collection database

Strain	No. of bp	BLASTn closest homolog (accession#) organism	Score	Coverage/ Identity [%]
TLT/PHB/001	1465	*Trichodesmium erythraeum* IMS101	2529	1433/1465 [97.8%]
		Trichodesmium sp.	2508	1425/1460 [97.6%]
		Okeania sp. PNG05-4	2503	1386/1401 [98.9%]
		Oscillatoria sp. PAB-21	2471	1356/1365 [99.3%]
TLT/PHC/001	1399	*Okeania* sp. PNG05-4	2490	1383/1400 [98.8%]
		Oscillatoria sp. PAB-21	2471	1356/1365 [99.3%]
		Oscillatoria nigroviridis 3LOSC	2471	1356/1365 [99.3%]
		Okeania sp. NAC8-18	2412	1335/1349 [99.0%]
TLT/PHC/002	1363	*Oscillatoria* sp. PAB-21	2451	1352/1364 [99.1%]
		Oscillatoria nigroviridis 3LOSC	2451	1352/1364 [99.1%]
		Okeania sp. PNG05-4	2423	1347/1364 [98.8%]
		Hydrocoleum glutinosum M34	2399	1331/1347 [98.8%]

Table S2. Complete 16s rRNA sequence of cyanobacterial samples

TLT/PHB/001

CTCAGGATGAACGCTGGCGGTCTGCTTAACACATGCAAGTCGAACGGACCCTTCGGGGTTAGTGGCGGACGGGTGAGTAA
CGCGTGAGAATCTGCCTTCAGGTCTGGGACAACAGAAGGAAACTTCTGCTAATCCCGGATGAGCCTTTGGGTAAAAGATA
AATTGCCTGGAGATGAGCTCGCGTCTGATTAGCTAGTTGGTGTGGTAAAAGCATACCAAGGCAACGATCAGTAGCTGGTC
TGAGAGGATGAGCAGCCACACTGGGACTGAGACACGGCCCAGACTCCTACGGGAGGCAGCAGTGGGGAATTTTCCGCAA
TGGGCGAAAGCCTGACGGAGCAAGACCGCGTGGGGGAGGAAGGCTCTAGGGTTGTAAACCCCTTTTCTTTGGGAAGAAG
TTCTGACGGTACCAAAGGAATCAGCCTCGGCTAACTCCGTGCCAGCAGCCGCGGTAATACGGAGGAGGCAAGCGTTATCC
GGAATGATTGGGCGTAAAGCGTCCGCAGGTGGCCATGTAAGTCTGCTGTCAAAACCCAGGGCTCAACTCTGGTCAGGCAG
TGGAAACTACAAAGCTAGAGTCTGGTAGGGGCAAAGGGAATTCCCGGCGTAGCGGTGAAATGCGTAGATTTCGGGAAGA
ACATCGGTGGCGAAAGCGCTTTGCTAGACCAGAACTGACACTCAGGGACGAAAGCTAGGGGAGCGAATGGGATTAGATA
CCCCAGTAGTCCTAGCCGTAAACGATGGATACTAGGTGTTGCCTGTATCGACCCAGGCAGTGCCGTAGCTAACGCGTTAA
GTATCCCGCCTGGGGAGTACGCACGCAAGTGTGAAACTCAAAGGAATTGACGGGGGCCCGCACAAGCGGTGGAGTATGT
GGTTTAATTCGATGCAACGCGAAGAACCTTACCAGGACTTGACATGTCGCGAATCTTAGGGAAACTTGAGAGTGCCTTCG
GGAGCGCGAACACAGGTGGTGCATGGCTGTCGTCAGCTCGTGTCGTGAGATGTTGGGTTAAGTCCCGCAACGAGCGCAAC
CCTCGTTTTTAGTTGCCATCATTAAGTTGGGCACTCTAAAGAGACTGCCGGTGACAAACCGGAGGAAGGTGGGGATGACG
TCAAGTCAGCATGCCCCTTACGTCCTGGGCTACACACGTACTACAATGGTCTGGACAGAGGGTAGCCAACCCGCGAGGGC
GAGCCAATCTCAAAAACCAGGCCTCAGTTCAGATTGCAGGCTGCAACTCGCCTGCATGAAGGAGGAATCGCTAGTAATCG
CAGGTCAGCATACTGCGGTGAATCCGTTCCCGGGCCTTGTACACACCGCCCGTCACACCATGGAAGTTGGCCACGCCCGA
AGTCATTACTCTAACTCGTAAGAGAGGGGGATGCCGAAGGCAGGGCTGATGACTGGGGTGAAGTCGTAACAAGGTAGCC
GTACCGGAAGGTGTGGCTGGATCACCTCCTT

TLT/PHC/001

GGACGGGTGAGTAACGCGTGAGAATCTGCCTTCAGGTCTGGGACAACAGAAGGAAACTTCTGCTAATCCCGGATGAGCCT
TTGGGTAAAAGATAAATTGCCTGGAGATGAGCTCGCGTCTGATTAGCTAGTTGGTGTGGTAAAAGCATACCAAGGCAACG
ATCAGTAGCTGGTCTGAGAGGATGAGCAGCCACACTGGGACTGAGACACGGCCCAGACTCCTACGGGAGGCAGCAGTGG
GGAATTTTCCGCAATGGGCGAAAGCCTGACGGAGCAAGACCGCGTGGGGGAGGAAGGCTCTAGGGTTGTAAACCCCTTTT
CTTTGGGAAGAAGTTCTGACGGTACCAAAGGAATCAGCCTCGGCTAACTCCGTGCCAGCAGCCGCGGTAATACGGAGGA
GGCAAGCGTTATCCGGAATGATTGGGCGTAAAGCGTCCGCAGGTTGCCATGTAAGTCTGCTGTCAAAACCCAGGGCTCAA
CTCTGGTCAGGCAGTGGAAACTACAAAGCTAGAGTCTGGTAGGGGCAAAGGGAATTCCCGGCGTAGCGGTGAAATGCGT
AGATTTCGGGAAGAACATCGGTGGCGAAAGCGCTTTGCTAGACCAGAACTGACACTCAGGGACGAAAGCTAGGGGAGCG
AATGGGATTAGATACCCCAGTAGTCCTAGCCGTAAACGATGGATACTAGGTGTTGCCTGTATCGACCCAGGCAGTGCCGT
AGCTAACGCGTTAAGTATCCCGCCTGGGGAGTACGCACGCAAGTGTGAAACTCAAAGGAATTGACGGGGGCCCGCACAA
GCGGTGGAGTATGTGGTTTAATTCGATGCAACGCGAAGAACCTTACCAGGACTTGACATGTCGCGAATCTCAGGGAAACT
TGAGAGTGCCTTCGGGAGCGCGAACACAGGTGGTGCATGGCTGTCGTCAGCTCGTGTCGTGAGATGTTGGGTTAAGTCCC
GCAACGAGCGCAACCCTCGTTTTTAGTTGCCATCATTAAGTTGGGCACTCTAAAGAGACTGCCGGTGACAAACCGGAGGA
AGGTGGGGATGACGTCAAGTCAGCATGCCCCTTACGTCCTGGGCTACACACGTACTACAATGGTCTGGACAGAGGGTAGC
CAACCCGCGAGGGCGAGCCAATCTCAAAAACCAGGCCTCAGTTCAGATTGCAGGCTGCAACTCGCCTGCATGAAGGAGG
AATCGCTAGTAATCGCAGGTCAGCATACTGCGGTGAATCCGTTCCCGGGCCTTGTACACACCGCCCGTCACACCATGGAA
GTTGGCCACGCCCGAAGTCATTACTCTAACTCGTAAGAGAGGGGGATGCCGAAGGCAGGGCTGATGACTGGGGTGAAGT
CGTAACAAGGTAGCCGTACCGGAAGGTGTGGCTGGATCACCTCCTT

TLT/PHC/002

TCTGGGACAACAGAAGGAAACTTCTGCTAATCCCGGATGAGCCGTGAGGTAAAAGAGAAATTGCCTGGAG
ATGAGCTCGCGTCTGATTAGCTAGTTGGTGTGGTAAAAGCATACCAAGGCAACGATCAGTAGCTGGTCTGA
GAGGATGAGCAGCCACACTGGGACTGAGACACGGCCCAGACTCCTACGGGAGGCAGCAGTGGGGAATTTT
CCGCAATGGGCGAAAGCCTGACGGAGCAAGACCGCGTGGGGGAGGAAGGCTTTAGGGTTGTAAACCCCTT
TTCTTTGGGAAGAAGTTCTGACGGTACCAAAGGAATCAGCCTCGGCTAACTCCGTGCCAGCAGCCGCGGTA
ATACGGAGGAGGCAAGCGTTATCCGGAATGATTGGGCGTAAAGCGTCCGCAGGTGGCCATGTAAGTCTGCT
GTCAAAACCCAGGGCTCAACTCTGGTCAGGCAGTGGAAACTACAAAGCTAGAGTCTGGTAGGGGCAAAGG
GAATTCCCGGCGTAGCGGTGAAATGCGTAGATTTCGGGAAGAACATCGGTGGCGAAAGCGCTTTGCTAGAC
CAGAACTGACACTCAGGGACGAAAGCTAGGGGAGCGAATGGGATTAGATACCCCAGTAGTCCTAGCCGTA
AACGATGGATACTAGGTGTTGCCTGTATCGACCCAGGCAGTGCCGTAGCTAACGCGTTAAGTATCCCGCCT
GGGGAGTACGCACGCAAGTGTGAAACTCAAAGGAATTGACGGGGGCCCGCACAAGCGGTGGAGTATGTGG
TTTAATTCGATGCAACGCGAAGAACCTTACCAGGACTTGACATGTCGCGAATCTTAGGGAAACTTGAGAGT
GCCTTCGGGAGCGCGAACACAGGTGGTGCATGGCTGTCGTCAGCTCGTGTCGTGAGATGTTGGGTTAAGTC
CCGCAACGAGCGCAACCCTCGTTTTTAGTTGCCATCATTAAGTTGGGCACTCTAAAGAGACTGCCGGTGAC
AAACCGGAGGAAGGTGGGGATGACGTCAAGTCAGCATGCCCCTTACGTCCTGGGCTACACACGTACTACAA
TGGTCTGGACAGAGGGTAGCCAACCCGCGAGGGCGAGCCAATCTCAAAAACCAGGCCTCAGTTCAGATTG
CAGGCTGCAACTCGCCTGCATGAAGGAGGAATCGCTAGTAATCGCAGGTCAGCATACTGCGGTGAATCCGT
TCCCGGGCCTTGTACACACCGCCCGTCACACCATGGAAGTTGGCCACGCCCGAAGTCATTACTCTAACCCGT
ATGGGAGGGGGATGCCGAAGGCAGGGCTGATGACTGGGGTGAAGTCGTAACAAGGTAGCCGTACCGGAAG
GTGTGGCTGGATCACCTCCTT
